# Screening of Fungal Strains and Formulations of *Metarhizium anisopliae* to Control *Phyllotreta striolata* in Chinese Flowering Cabbage

**DOI:** 10.3390/insects14060567

**Published:** 2023-06-19

**Authors:** Wei Chen, Wenjing Yuan, Renkun He, Xinhua Pu, Qiongbo Hu, Qunfang Weng

**Affiliations:** National Key Laboratory of Green Pesticide, College of Plant Protection, South China Agricultural University, Guangzhou 510642, China; cw@stu.scau.edu.cn (W.C.); ywj1078034776@163.com (W.Y.); m18702033809@163.com (R.H.); pu123xh@163.com (X.P.)

**Keywords:** *Phyllotreta striolata*, *Metarhizium anisopliae*, *Brassica campestris*, seed pelletization, mixed insecticide

## Abstract

**Simple Summary:**

The cabbage flea beetle (CFB; *Phyllotreta striolata*) is a significant pest of cruciferous vegetables in the world. CFB causes severe destruction of Chinese flowering cabbage (CFC; *Brassica campestris* L. ssp. *chinensis* var. *utilis*), a key leafy vegetable in South China. In practice, the current control approach of CFB is to kill adults by spraying chemical insecticides, but the control efficacy is not sufficient because the adults have a strong tolerance to insecticides; on the other hand, the larvae living in soil are not influenced by pesticides. It is urgent to develop sustainable technology to control CFB. Here, we provide a new technology to control larvae and adults through seed pelletization and stem–leaf spraying using *Metarhizium anisopliae*. This study provides new insights into the application of mycoinsecticides and the IPM of flea beetle.

**Abstract:**

(1) Background: The cabbage flea beetle (CFB; *Phyllotreta striolata*) seriously damages the production of Chinese flowering cabbage (CFC; *Brassica campestris* L. ssp. *chinensis* var. *utilis*), which is a key leafy vegetable in South China. A large number of chemical insecticides have been sprayed to control this pest; as a result, residues and resistances are becoming an issue. It is necessary to develop biocontrol technologies to address this issue. (2) Methods: Fungal strains were selected based on bioactivity against CFB, and CFC seed pelletization with fungal conidia was subject to evaluation of control efficacy against CFB. The effective mixture of fungus and chemical insecticide was determined based on safety and joint toxicology tests. (3) Results: The screening of 103 strains from 14 genera identified the *Metarhizium anisopliae* strain MaGX19S02 (Ma) as the one with the highest virulence. The LC_50_s of Ma to CFB adult and second instar larvae on day 9 post-treatment were 3.04 × 10^6^ and 27.2 × 10^6^ spores/mL, respectively. In the pot test, the pelletization of CFC seeds with Ma conidia (50/25/12.5 mg in 1 g seed with 4 g fillers) demonstrated significant CFB mortalities (45–82%) 20 days after the larvae were introduced. In the field test, the seed pelletization achieved 57–81% control efficacy 14 days after sowing. Furthermore, the combination of Ma with chlorfenapyr (Chl) demonstrated a synergistic effect against CFB; based on this result, we prepared the mixture formulation of 20% Ma-Chl wettable powder (WP). The assessment of the effects of 20% Ma-Chl WP (500× diluent) against CFB revealed 93.33% mortality in the pot test and 61.3% control efficacy in the field test on day 7 post-treatment. (4) Conclusions: The findings demonstrate the potential of Ma to control CFB in the field. Seed pelletization with Ma conidia effectively controlled CFB larvae and protected CFC seedlings, wherein a mixture formulation of 20% Ma-Chl WP had substantial efficacy in controlling CFB adults. Our research provides new methods for CFB biocontrol.

## 1. Introduction

The cabbage flea beetle (CFB; *Phyllotreta striolata* (Fab.), Coleoptera: Chrysomelidae) is a major pest of cruciferous plants worldwide [1,2]. CFB has emerged as a disaster since 1990 in cruciferous leafy vegetables, such as Chinese flowering cabbage (CFC; *Brassica campestris* L. ssp. *chinensis* var. *utilis* Tsenet Lee) in South China [3]. CFB is highly tolerant to environmental stresses, including chemical pesticides, and surpasses the phyto-immunity exerted by the glucosinolate–myrosinase system, the so-called “mustard-oil bomb” in cruciferous plants [4]. The life cycle of CFB comprises four stages: egg, larva, pupa (below ground), and adult (above ground). The larvae infest the roots of the plants and seed buds leading to seedling deficiency, while the adults feed on leaves to decrease vegetable production and quality. In practice, the adults are preferred for control methods because the larvae are covert. However, the adults have a strong tolerance to chemical pesticides [5]. In China, there are approximately thirty pesticides (single and mixture) registered (http://www.chinapesticide.org.cn/zwb/dataCenter, accessed on 2 June 2023), but the most common insecticides such as organophosphorus, pyrethroids, and neonicotinoids only have a control efficacy of <85%, based on our evaluation (no published). Therefore, larger consumption of pesticides is required to control CFB, consequently leading to food safety and environmental pollution issues [2].

Therefore, developing sustainable strategies involving biological control agents (BCA) to regulate the population of CFB is of immense practical importance. The potential of entomopathogenic nematodes, fungi, bacteria, and parasitoids as BCAs has been rigorously investigated at the laboratory scale. Recent research reported that *M. anisopliae* strain Ma6 is highly pathogenic against CFB with a 7-day cumulative mortality rate of 91.11% and a median lethal time (LT_50_) of 4.09 days [6]. While both nematode species *Steinernema carpocapsae* and *Heterorhabditis indica* were capable of reducing populations of the soil-dwelling stages of CFB [7]. However, their large-scale application in the fields is limited [7,8,9], which could be attributed to the difficulty in developing BCAs, low efficacy, and higher costs. Furthermore, several factors, such as strain/trait faults and unsuitable formulation or application, also lead to low efficacy.

Entomopathogenic fungi (EF), such as *M. anisopliae* and *Beauveria bassiana*, are soil-dwelling fungi that colonize the rhizosphere and promote plant growth [10,11]. Several studies have demonstrated the efficacy of EF in controlling underground and aboveground pests worldwide. The fungal conidia are prepared in various formulations, including granules, dust powder, wettable powder (WP), oil suspension, and oil-dispersed suspension to control underground and leaf pests through soil treatment or spraying delivery [12,13,14]. However, the reports on the application of EF against CFB are limited. In this study, we aimed to test the potential of different formulations of EF to control CFB. Our findings unravel the potential of seed pelletization with *M. anisopliae* conidia and a mixture formulation of *M. anisopliae* with chlorfenapyr (Chl) to control CFB in CFC.

## 2. Materials and Methods

### 2.1. Fungi and Insects

A total of 103 fungal strains preserved in our laboratory were used. The conidia were inoculated onto the PDA plates from slants. After being cultured for two weeks at 26 °C, the conidia were collected and suspended with 0.05% Tween-80 solution and calibrated to desired concentration following the hemocytometry.

The *P. striolata* population was reared with CFC for more than five generations in a greenhouse at 26 °C maintained with a photoperiod of 14:10 h (light:dark). To collect larvae, adults were moved to a box laid with paper for oviposition—the eggs with the paper were transferred to a box with radish (*Raphanus sativus*) pieces cultured in an artificial climate chamber (LRH-800A-GSI, Guangdong Medical Equipment Co., Ltd., Shaoguan, China) at 26 ± 1 °C with a photoperiod of 14:10 h (light:dark) and 100% humidity—for egg hatching and larval feeding. Adults and larvae with consistent growth were selected for biological activity assays.

### 2.2. Bioassay of Fungal Strains against CFB in the Laboratory

The bioactivities of fungal strains to CFB were tested using the immersion method (referring to China Agricultural Standard NY/T 1154.6). After the adult *P. striolata* were anesthetized with CO_2_, they were moved into the tubes filled with conidial suspension with 1 × 10^7^ spores/mL and immersed for 20 s. Afterward, they were transferred to a Petri dish lined with filter paper, fed with fresh CFC leaves, and placed in an artificial climate chamber at 26 ± 1 °C with a photoperiod of 14:10 h (light:dark) and 100% humidity. The 0.05% Tween-80 solution was used as a control group. The experiment was replicated 3 times, and 10 insects in each treatment were used. 

The strain MaGX19S02 (Ma, with a GDMCC access NO. 60935) with the best bioactivity was further tested to determine the LC_50_ and LT_50_ to CFB adults and 2nd instar larvae. The adults were treated with serial suspensions of Ma conidia with the described concentration. For the treatment of larvae, first, the radish (*Raphanus sativus*) pieces were dipped in the conidial solution for 10 s and dried, then, larvae (n = 10) were moved onto the radish pieces and placed in an artificial climate chamber at 26 ± 1 °C in the dark, and the old radish pieces were substituted with fresh ones every day. There were 10 insects in each treatment and the experiments were repeated four times. The diseased insects were determined based on the presence of the fungal colony on the cadaver. 

### 2.3. Pelletization of CFC Seeds with Ma Conidia

#### 2.3.1. Preparation of Pelletized CFC Seed

The conidia of Ma were collected from the rice substrates (inoculated with fungal hyphal broth and cultured at 26 °C for 2 weeks) using a spore harvester (BFQ100, Chishun Tech, Nanjing, China). The conidia powder was dried in a freeze dryer (BiLon, Shanghai, China), and then sealed and stored at 4 °C. Before use, the key quality indicators of conidia powder were determined as conidia number (2.36 ± 0.06) × 10^10^/g, life conidia (96.69 ± 0.92)%, and water content (2.15 ± 0.13)% [15].

CFC seeds (provided by Jianfeng Seeds, Guangzhou, China) and filler (provided by Guangdong Plant Protection Research Institute, Guangzhou, China) at a ratio of 1:4 (m:m) were used for pelletization. The conidia powder doses were, respectively, designated as 12.5, 25, and 50 mg/g seed. A coating machine (Niklas W.N.5/01, Willy Niklas GmbH Apparatebau, Moenchengladbach, Germany) was employed to prepare pelletized seeds. The quality control of pelletized seeds was ensured by referring to the China National Standards (GB/T 25240, GB/T 5520) and the China Agricultural Standard (NY/T 1965.1). 

#### 2.3.2. Bioassay of Pelletized Seed against CFB in Pot Tests

Plastic pots (80 × 100 mm) were used for pot tests. Each pot was filled with general nutrient soil (100 g, Huanuo Biotech, Shengyang, China). The pelletized seeds (n = 30) were sowed in each pot. A total of 10 1st instar larvae were introduced into each pot. Then, the pots were incubated in an artificial climate chamber at 26 °C with a photoperiod of 14:10 h (light:dark) and a relative humidity of 100%. A total of 3 biological replicates were carried out with 10 insects for each dose treatment. Each experiment was repeated three times. The filler pelletized seeds (no conidia and other active ingredients) were used as a control. The seedling rates (seedlings/seeds), plant height, and insect mortality were investigated after treatment. 

#### 2.3.3. Efficacy Evaluation of Pelletized Seed against CFB in Fields

The field trials were conducted at the South China Agricultural University (Guangzhou, China) farm in two seasons from September–November 2020 and February–April 2021, following a random block design with three replications. Pelletized seeds of 40 g or normal seeds of 20 g were sown to each plot (20 m^2^). Ma treatments of 50, 25, and 12.5 mg/g of seed were applied, while filler pelletized seeds and normal seeds were used as the control. The plots were covered with nylon mesh after treatment. The control efficacy (CE) of the fungal formulations was evaluated based on the DI of CFC by referring to the China National Standards (GB/T 17980.18):

$$DI=\frac{\Sigma (Ni\times i)}{\left(T\times 9\right)}\times 100,$$

where DI is the leaf damage index, Ni is the number of leaves with i level, and T is the total number of leaves investigated;

$$CE(\%)=(1-\frac{CK1\times PT2}{CK2\times PT1})\times 100,$$

where CE is the control efficacy, CK1 is the DI in the control group before treatment, CK2 is the DI in the control group after treatment, PT1 is the DI in the treated group before treatment, and PT2 is the DI in the treated group after treatment.

### 2.4. Mixture of Ma with Chemical Insecticides

#### 2.4.1. Biosafety of Chemical Insecticides to Ma in Lab

Nine technical-grade chemical insecticides were used: 97% Chl and 97% tolfenpyrad (Qianjiale Biotech, Guangzhou, China); 97% dinotefuran, 70% emamectin benzoate, 97% diafenthiuron, and 96% acetamiprid (Yinhui CropSci, Foshan, China); and 99% pyridaben, 95% rotenone, and 97% thiamethoxam (Macklin Biochem, Shanghai, China). The effects of these insecticides on Ma conidia germination and mycelial growth in shaking culture and PDA plate were investigated (referring GB/T 25864). The insecticides were dissolved into 10,000 mg/L (stock solution) of acetone and diluted to 10 and 100 mg/L with 0.05% Tween-80 (working solutions). The solution of 100 mg/L acetone with 0.05% Tween-80 was used as a control. The experiment was repeated three times. The live conidia and colony diameter were investigated after treatment.

#### 2.4.2. Bioassay of Ma with Chl and Preparation of Mixture WP

The bioactivities of Ma, Chl, and their mixture to CFB adults were assessed using the insect immersion method (NY/T 1154.6). The CFB adults were treated with a serial concentration of Ma, Chl, and their mixture. The number of diseased and dead insects was assessed after the treatment following the steps described in Section 2.2. A total of 3 biological replicates were carried out with 10 insects for each dose treatment. Each experiment was repeated three times.

The mixture WP of MaGX19S06 conidia and Chl was prepared following the China National Standards GB/T5451, GB/T14825, GB/T 1601, and GB/T 16150. 

#### 2.4.3. Bioassay of Ma-Chl WP against CFB in Pot Tests

Fifty seeds were sowed in each pot. After 20 days of culture, each pot was sprayed with different concentrations of Ma-Chl WP (10 mL). Then, CFB adults (n = 10) were released into each pot, and the pots were covered with a nylon mesh to prevent insect escape. Tap water was used as a control. The mortality rate of adults and the damage index (DI) of CFC were assessed according to the China National Standards (GB/T 17980.18). The experiment was repeated three times. The other experiment procedures were the same as described in Section 2.3.2.

#### 2.4.4. Efficacy Evaluation of Fungal Formulations against CFB in Fields

The plots were treated with 400 mg/L Ma-Chl WP (20% Ma-Chl WP diluted 500×), 50 mg/L Chl, and 0.05% Tween-80 solution (control, CK). Each plot was sprayed with 1 L of diluent when the CFC adult density was about 1 per plant. The other experiment procedures were the same as described in Section 2.3.3.

### 2.5. Statistical Analysis

The DPS (Hangzhou Ruifeng Information Technology Co., Ltd., Hangzhou, China) software was employed to conduct the statistical analyses of the data [16]. The treatment and control means were compared using one-way ANOVA followed by Duncan’s multiple range test (DMRT) analyses or *t*-test. The LC_50_ and LT_50_ values were evaluated using probit regressions. The joint virulence was calculated using Sun’s co-toxicity coefficient (CTC) [17]. 

## 3. Results

### 3.1. Bioactivity of Fungal Strains against CFB

The results of bioactivities of the 103 fungal strains against *P. striolata* are shown in Table S1. The mortality of CFB adults ranged from 0 to 70% after treatment with conidia suspension (1 × 10^7^ spores/mL) of different strains, and the highest bioactivity (46–53%) was achieved with the *M. anisopliae* strain, MaGX19S02, on days 9–11 after treatment. 

**Figure 1:**
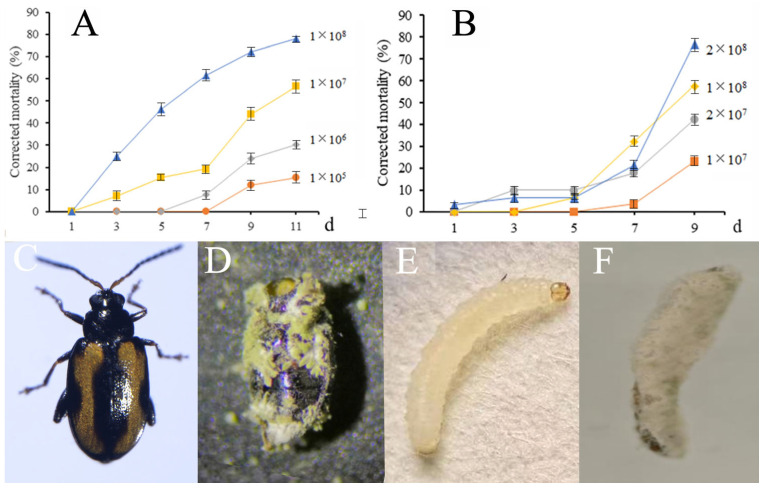
Bioactivity of Ma against CFB. The mortality of CFB adults (**A**) and 2nd instar larvae (**B**) treated with Ma. Morphological characteristics of CFB adult in control (**C**), adult cadaver (**D**) on day 11 after treatment with Ma (the beetle body was covered by green Ma conidia), larva in control (**E**), and larva cadaver (**F**) on day 11 after treatment with Ma (the larva body was covered by Ma hyphae and conidia). The insect soaking method was used to conduct the adult bioassay. The anesthetized insects with CO_2_ were moved into the tubes filled with conidial suspension with a designated conidial concentration and immersed for 20 s, placed in an artificial climate chamber at 26 ± 1 °C with a photoperiod of 14:10 h (light:dark) and 100% humidity, and fed with fresh CFC leaves every day. The experiment was replicated 3 times, and 10 insects in each treatment were used. For the larvae bioassay, first, the radish (*R. sativus*) pieces were dipped in the conidial solution for 10 s and dried. Afterward, larvae (n = 10) were moved onto the radish pieces and placed in an artificial climate chamber at 26 ± 1 °C in the dark, and the old radish pieces were substituted with fresh ones every day. There were 10 insects in each treatment and the experiments were repeated 4 times. After the treatments, the diseased insects were determined based on the presence of the fungal colony on the cadaver. A increased mortality was demonstrated in CFB adults and larvae in a dose- and time-dependent manner.

Next, we assessed the virulence of MaGX19S02, which demonstrated increased mortality in CFB adults and larvae in a dose- and time-dependent manner (Figure 1). At 1 × 10^8^ spores/mL, the corrected mortalities of adults and larvae were up to 60–70% on days 9 and 11 post-treatment; the corpses of the insects showed apparent symptoms of fungal infections (Figure 1). At the same concentration, the pathogenicity was higher in adults than that in larvae, which could be because of the inhibiting effect of fresh radish on fungal growth. MaGX19S02 was preserved at the Guangdong Microbial Culture Collection Center (Guangzhou, China) with an access number GDMCC 60935.

The LC_50_ values of MaGX19S02 against CFB adult and second instar larvae on day 9 were 3.04 × 10^6^ and 27.2 × 10^6^ spores/mL, respectively. The LT_50_ values of Ma at 1 × 10^8^ spores/mL against adult and second instar larvae were 4.9 and 9.3 days (Table 1). 

### 3.2. Effects of Ma Conidia Pelletized Seeds on CFB

#### 3.2.1. Efficacy of Pelletized Seeds in the Pot Tests

**Figure 2:**
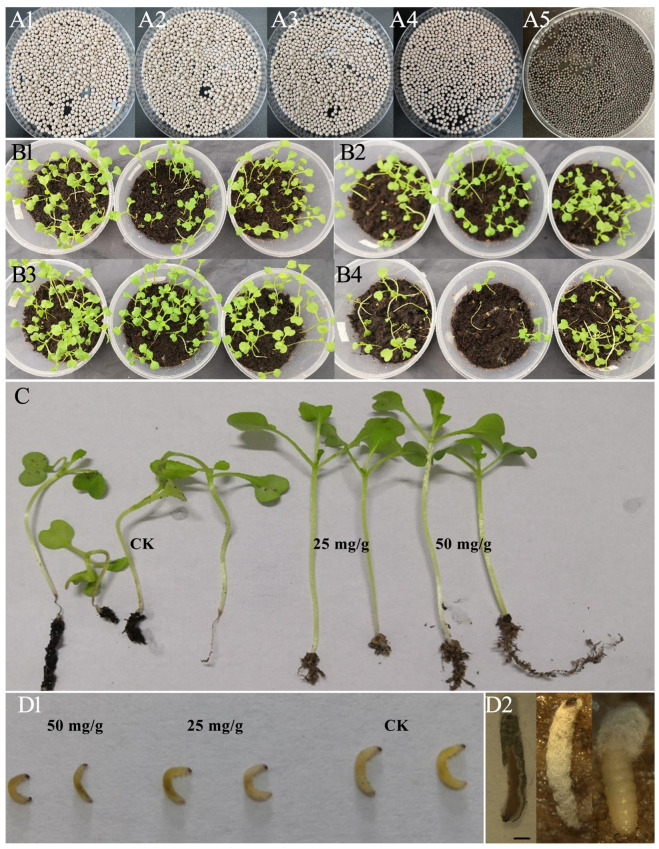
Control efficacy of CFC seed pelletization with Ma conidia in the pot test. (**A**) Profiles of CFC pelletized seeds with Ma conidia 12.5 (**A1**), 25 (**A2**), and 50 (**A3**) mg in each g seed (**A4**), filler pelletized seeds; (**A5**), normal seeds; CFC seed and filler at a ratio of 1:4 (m:m). A coating machine was employed to prepare the pelletized seeds. The quality control of the pelletized seeds was ensured by referring to the China National Standards (GB/T 25240, GB/T 5520) and the China Agricultural Standard (NY/T 1965.1). (**B**) Seedling profiles of CFC seeds pelletized with Ma conidia at 12.5 (**B1**), 25 (**B2**), and 50 (**B3**) mg/g seed, and the control (**B4**) (filler pelletized seeds) in the pot test on day 20 post-sowing. (**C**) The seedlings were higher and stronger in the treatment group of 50 and 25 mg/g of seed than in the control group (CK) on day 20 post-sowing. (**D**) The CFBs were smaller and presented infected spots in the treatment group compared with the control group (CK) on day 7 post-sowing (**D1**), and larvae cadavers in treatment died by Ma on day 14 post-sowing (**D2**), bar = 1 mm. In each pot, 30 seeds were sown and 10 1st instar larvae were introduced.

The CFC seeds were pelletized (Figure 2A). The pot test demonstrated the potential of Ma conidia pelletized seeds to control CFB larvae and improve the CFC seedling rate and plant height (Table 2, Figure 2). The CFC seedling numbers were apparently higher in the treatment groups than in the control (Figure 2B). Moreover, the seedlings from the treatment groups looked stronger and had better roots than the control (Figure 2C), while the larvae in the treatment groups, but not in the control, were smaller with brown spots on the cuticle on day 7 post-treatment (Figure 2D). The CFB cadavers with Ma mycelia and conidia were also found in all groups other than in the control on day 14 post-treatment (Figure 2D).

After 20 days of treatment, the mortality of the CFB larvae increased with an increased conidial dose of Ma. The corrected mortality rates of the larvae were significantly higher in Ma pelletized seeds (45.45–81.82%) than that in the control (3.33%) (Table 2). The CFC plant rates and heights were also significantly higher in treatments than those in the control (Table 2). 

#### 3.2.2. Efficacy of Pelletized Seeds against CFB in Fields

**Figure 3:**
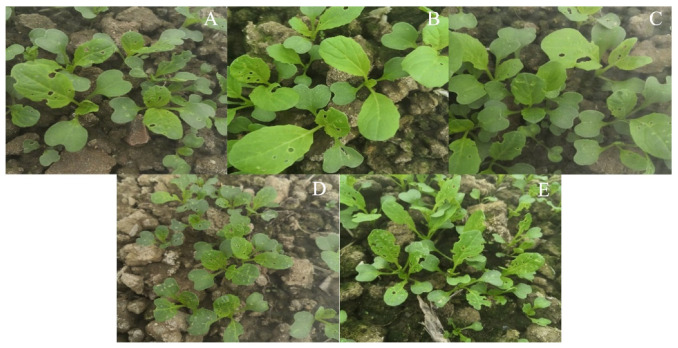
Seedling profiles of CFC seeds pelletized with or without MaGX19S02 conidia in the field test on day 14 post-sowing. MaGX19S02 conidia pelletized seeds at 12.5 (**A**), 25 (**B**), and 50 (**C**) mg/g seed, control (**D**) filler pelletized seeds, and (**E**) normal seeds. The field trials were conducted on a farm at the South China Agricultural University (Guangzhou, China) in 2020–2021. The trial followed a random block design. Each plot measured 20 m^2^ and 40 g of pelletized seeds were sowed (containing 8 g of normal seed, 32 g fillers, and active ingredients) or 20 g of normal seeds. The experiment was repeated three times. The efficacy was evaluated based on the damage index of the CFC by referring to the China National Standards (GB/T 17980.18). is the results indicate that CFCs in the treatment group (A/B/C) had fewer holes in leaves and higher height than those in the control group (D/E).

On days 7–14 post-treatment, the CFC DI and control efficacy (CE) in Ma pelletized seeds were 1–16% and 41–93%, respectively, which were significantly better than those in the filler pelletized seeds (8.7–40.7% and 33%, respectively). The DI in normal seeds was also significantly higher (13.2–37.0%) than that in the Ma pelletized seeds (Table 3). Consistent with the results of the pot test, the CE in Ma pelletized treatments at 12.5 mg/g seed was significantly lower than those in the other two doses, wherein the CE in 25 and 50 mg conidia/g seed did not differ significantly (Table 3). Similar effects on the growth profiles of the CFC plants were observed; the number of healthy plants in plots sown with Ma pelletized seeds was higher than those in plots sown with filler and control pelletized seeds (Figure 3).

### 3.3. Effects of the Fungal–Chemical Mixture against CFB

#### 3.3.1. Biosafety of Chemical Insecticides to MaGX19S02 in Lab

The effects of nine chemical insecticides on conidia germination and mycelia growth of Ma are shown in Table 4. The data showed that diafenthiuron, dinotefuran, pyridaben, rotenone, and tolfenpyrad had side effects on Ma conidia germination and mycelial growth. However, the other four chemicals, acetamiprid, Chl, emamectin benzoate, and thiamethoxam, did not decrease the conidia germination and mycelial growth, amongst which Chl showed the best biosafety to Ma. Therefore, Chl was selected to prepare a mixture formulation with Ma.

#### 3.3.2. Virulence of Ma-Chl Mixture against CFB in the Laboratory

**Figure 4:**
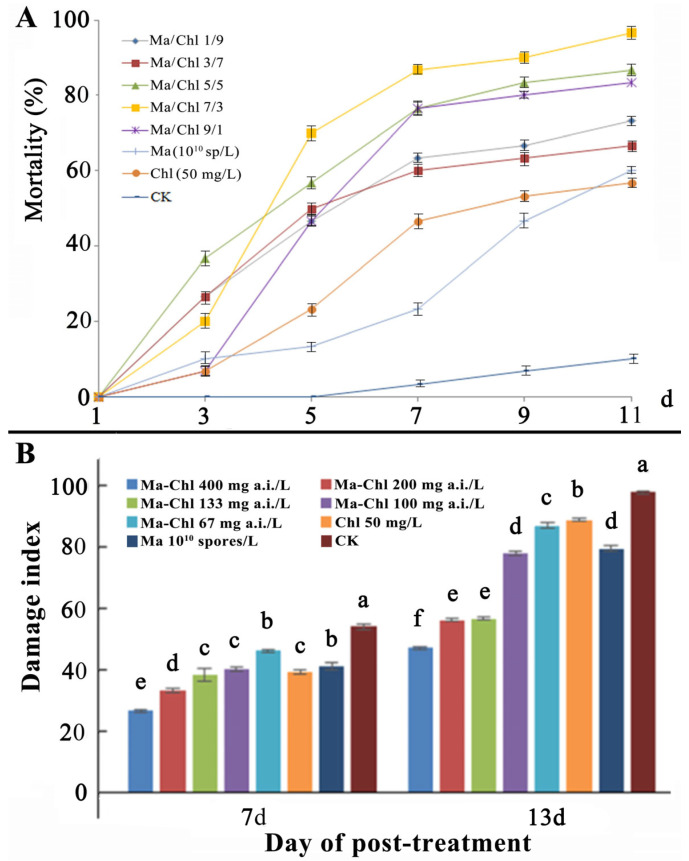
(**A**) Effect of the Ma-Chl mixture on the mortality of the CFB adults in the laboratory bioassay. Ma/Chl represent the volume ratio of Ma and Chl. The insect soaking method was used. The experiments were repeated 3 times with 10 insects in each treatment. (**B**) The CFC damage index in pot tests using 20% Ma-Chl WP. Different letters indicate significant differences (*p* < 0.05) in the DMRT. A total of 50 seeds were sowed in each pot. After 20 days of culture, each pot was sprayed with different concentrations of Ma-Chl WP (10 mL). Then, CFB adults (n = 10) were released into each pot, and the pots were covered with a nylon mesh to prevent insect escape. Tap water was used as a control. The mortality rate of adults and the damage index (DI) of the CFC were assessed according to the China National Standards (GB/T 17980.18). The experiment was repeated three times.

The curves of dosage–time–mortality of Ma, Chl, and their mixtures are shown in Figure 4A. In total, the Ma-Chl mixture (A/B) increased the mortality of CFB adults in a dose-dependent manner (Figure 4). In particular, the 7/3, 5/5 and 9/1 mixtures of Ma/Chl (Ma 10^10^ spore/L, Chl 50 mg/L) had higher mortalities than the single Ma or Chl on days 7–11 post-treatment (Figure 4A).

Furthermore, log-probit regression analysis indicated that the Ma-Chl mixture had lower LT_50_ values than single treatments. In particular, the LT_50_ values of the Ma-Chl mixture at active ingredient (a.i.) rates of 212/25 (5/5), 296/15 (7/3), and 381/5 (9/1) were 5.19, 4.82, and 6.26 days, which had 151.59, 204.18, and 209.86 CTCs (Table 5), suggesting that the Ma and Chl at these three rates exert synergistic effects. Therefore, for further experiments, we prepared the 20% (m/m) mixture WP (20% Ma-Chl WP, including 19% Ma conidia, 1% Chl, 80% filler, and other adjuvants). 

#### 3.3.3. Efficacy of the Ma-Chl WP against CFB in Pot Test

On day 7 post-treatment, 20% Ma-Chl WP 500× diluent (400 mg a.i./L) had the highest mortality (93.33%) of CFB, which was significantly higher than the other treatments. In addition, the lower concentration (1000× and 1500×) treatment groups of Ma-Chl WP were more effective than Chl or Ma alone (Table 6).

As shown in Figure 4B, the DI in three Ma-Chl WP treatments at 400, 200, and 133 mg a.i./L ranged from 25–40 on day 7 and 47–57 on day 13 post-treatment, which were significantly lower than those in the other treatments and controls. 

**Figure 5:**
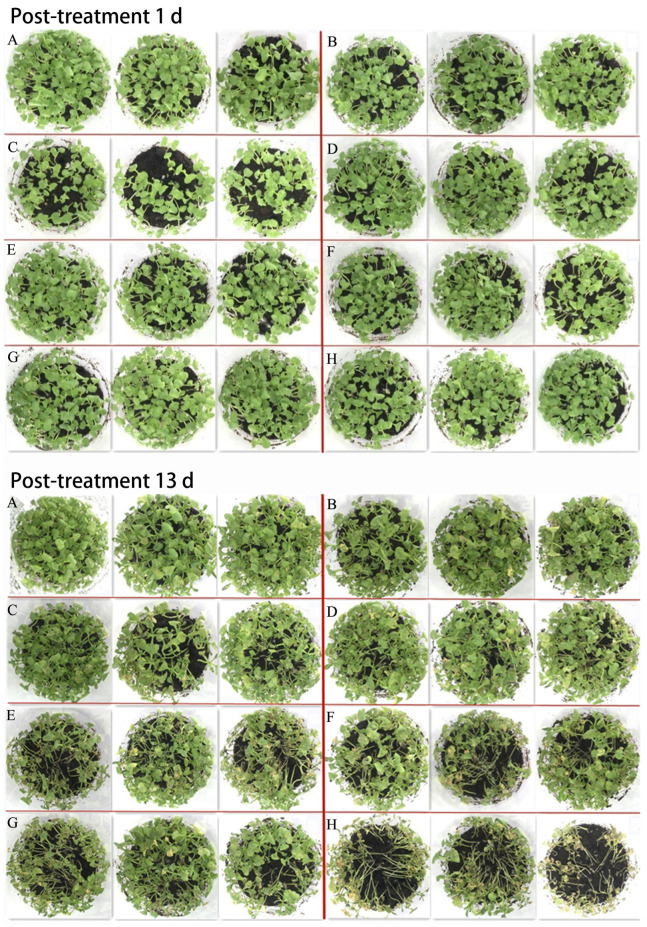
The profiles of CFC growth in the pot tests using 20% Ma-Chl WP. (**A**–**E**) 20% Ma-Chl WP at 400, 200, 133, 67, and 50 mg a.i./L, respectively; (**F**) chlorfenapyr 50 mg/L; (**G**) MaGX19S02 at 10^10^ spores/L (424 mg/L); (**H**) CK (control, tap water). A total of 50 seeds were sowed in each pot. After 20 days of culture, each pot was sprayed with different concentrations of Ma-Chl WP (10 mL). Then, CFB adults (n = 10) were released into each pot, and the pots were covered with a nylon mesh to prevent insect escape. Tap water was used as a control. The mortality rate of adults and the damage index (DI) of the CFC were assessed according to the China National Standards (GB/T 17980.18). The experiment was repeated three times.

Similar effects of Ma-Chl WP at 400, 200, and 133 mg a.i./L were observed on the growth profiles of CFB plants (Figure 5). On day 13 post-treatment, the CFB-induced damage in the seedlings in these three treatment doses was low compared with those in the seedlings in the control and other treatment doses. Moreover, wilting and moldiness were observed in the control pots (Figure 5).

#### 3.3.4. Efficacy of Ma-Chl WP against CFB in Fields

As shown in Table 7, the DI and CE values in the Ma-Chl WP group (19.4–24.4% and 61.3–56.2%, respectively) were significantly different than those in the Chl group (56.7–84.4% and 24.8–(-)0.9%, respectively) on days 7–14 post-treatment.

**Figure 6:**
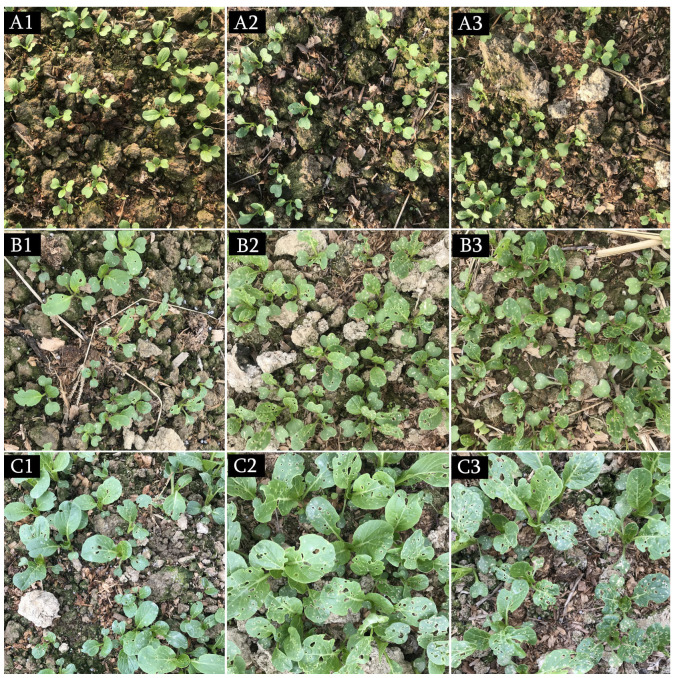
The profiles of CFC growth in the field tests using 20% Ma-Chl WP. (**A1**–**C1**), Ma-Chl 400 mg a.i./L on days 1 (**A1**), 7 (**B1**), and 14 (**C1**) post-treatment; (**A2**–**C2**), Chl 50 mg a.i./L on days 1 (**A2**), 7 (**B2**), and 14 (**C2**) post-treatment; **A3**–**C3**, control (CK) on days 1 (**A3**), 7 (**B3**), and 14 (**C3**) post-treatment. The field trials were conducted at the South China Agricultural University (Guangzhou, China) farm in 2020–2021, following a random block design with three replicates. Each plot (20 m^2^) was sown with 20 g of normal seeds and sprayed with 1 L of diluent when the CFB adult density was ~1/plant. The plot was covered with nylon mesh after spraying. The efficacy was evaluated based on the damage index of the CFC referring to the China National Standards (GB/T 17980.18).

As shown in Figure 6, on days 1–14 post-treatment, the damage rates of CFC plants in the fields treated with Ma-Chl WP were lesser than those in the fields treated with Chl or CK.

## 4. Discussion

The CFB has been prevailing since the 1990s in Southern China with the popularity of vegetables of the mustard family [18]. Although CFB has several host plants in the mustard family, such as *R. sativus*, *B. oleracea, B. napus*, and *B. rapa*, it prefers CFC [19]. In addition, the continuous replanting of CFC (replanted 5–8 times every year in South China) provides the best food to CFB. The characteristic life cycle and the occurrence of CFB protect it against environmental and human interferences and defend it from the immunity of crucifer plants [4]. In recent years, research on the control of CFB has received increasing attention. It was shown that entomopathogenic nematodes *S. pakistanense* 94-1 and *H. indica* 212-2 inhibit CFB larvae at a concentration of 10^9^ or higher in infected juveniles [20]. Chen et. al silenced the dre4 gene using the technique of RNA interference and found that the silencing of dre4 contributed to the high mortality of CFB [21]. CFB adults have active, alert features and high tolerance to pesticides and live on the ground to feed on leaves, while the larvae hide in the soil and infest the seeds and roots. Like larvae, the eggs and pupae live in the soil, although they do not damage crops; all these factors cumulatively make the control of CFB difficult. Therefore, we think that the key strategies to control CFB should be based on soil treatment to decrease the overall population growth of CFB, seed treatment to protect the buds and seedlings from CFB infestation, stem–leaf treatment to prevent CFB migration, and IPM to promote better growth and harvest.

In this study, we reported the efficiency of seed and stem–leaf treatment strategies employing an EF BCA, *M. anisopliae*, to manage CFB and improve CFC production. The efficiencies of BCAs, such as *M. anisopliae,* have been studied for a long time on the laboratory scale, identifying several strains causing >50% mortality in the CFB [6]; however, only a few commercial products based on these strains as a soil treatment or stem–leaf spraying treatment are used in fields.

For the first time, our study explored seed pelletization with Ma to control CFB. Usually, seed treatment requires systemic insecticides such as neonicotinoids. It has been shown that fipronil has better efficacy in seed treatment than thiamethoxam against CFB [22]; however, fipronil and some neonicotinoids are forbidden or only permitted for limited application in most regions of the world. Crucifer plants, especially *Brassica* spp., have small seeds, which limits the drug loading of conventional seed coating and affects the CE against CFB. Indeed, pelletization can increase the drug loading on the surface of the seed, accommodating enough conidia for colonization of Ma and forming a protective sphere to prevent CFB infestation of the buds and roots. As is evident from the symptoms of mycosis demonstrated in the pot tests, Ma can infect CFB larvae upon its entrance into the fungal sphere, consequently preventing CFB infestation in CFC.

It is possible that the control efficacy of Ma pelletization against CFB is owing to insecticidal compounds or phyto-immunity induced by Ma fungal endophyte stimulation. In fact, multi-nutrient interactions often exist in entomopathogenic fungi used for biological control [23,24]. *M. anisopliae* colonizes the plant rhizosphere and is also an endophytic fungus [23]. Through endophytes, *M. anisopliae* not only promotes cucumber, corn, and tomato to grow better and defend against pathogens [25,26], but it also changes the composition and distribution of nutrients such as amino acids, and forms new antibiotics or other secondary compounds in plants [27]. Therefore, it is a rational inference that in this study the effects of Ma pelletization partly originate from fungal endophytes resulting in a larger defense of CFC. From the field trial, it was observed that seed pelletization with Ma decreased the leaf damage levels, which suggests that CFB adults are controlled, although the adults do not directly come into contact with Ma. This effect could be related to the efficacy of Ma against the underground larvae, pupae, and egg mortality, consequently decreasing the adult density. Another reason might be that the adults are infected by Ma, although a few moldy cadavers were found in the field. Furthermore, seed pelletization can reduce Ma conidia consumption and decrease the cost. For example, soil treatment using granule formulation (for example, 200 × 10^6^ spore/g CQMa421 granule) every time consumes (10–20) × 10^12^ spores/hm^2^, while seed pelletization needs (3–5) × 10^12^ spores/hm^2^ with better CE. Together, the present study provides sufficient experimental evidence that Ma can be used for seed treatment through pelletization to control CFB.

Several studies have reported the combination synergisms of Ma with chemicals. For example, Ma and chlorantraniliprole exhibited a synergistic effect against *Spodoptera litura* larvae [28], Ma and imidacloprid were synergistic against *Cyrtomenus bergi* nymphs [29], while Ma and hydramethylnon had synergism with German cockroaches [30]. This study is the first to demonstrate the synergism of Ma and Chl against CFB adults. The formulation of 20% Ma-Chl WP was prepared because the Ma/Chl rate of 296/15 can prominently shorten the LT_50_ and has a lower cost and dosage of the a.i. In field application, a dose of 200 g a.i. per hm^2^ (1 kg 20% Ma-Chl WP, diluting 500×) is required, the cost of which is almost equal to that of 10% Chlorfenapy SC (500 g a.i., or 500 L of 1000× diluent/hm^2^, 130 RMB/hm^2^); this suggests the low cost of a mixed formulation of Ma-Chl WP for CFB control.

## 5. Conclusions

In conclusion, the findings of this study demonstrate that seed pelletization with the conidia of the MaGX19S02 strain, which showed the highest bioactivity against CFB among the tested 103 strains of EF, can effectively control CFB larvae and protect CFC seedlings. Furthermore, the study provides evidence that MaGX19S02 with Chl exerts a synergistic effect against CFB. The results suggest that the formulation of 20% Ma-Chl WP has substantial efficacy in controlling CFB adults. Our research provides new insights into CFB biocontrol on CFC in the field.

## Figures and Tables

**Table 1:** Virulence of MaGX19S02 against CFB.

CFB	LC_50_ (95% Confidence Interval, ×10^6^ Spore/mL)	LT_50_ (95% Confidence Interval, d)
Adult (9 d post-treatment)	3.04 (0.72–5.79)	
2nd instar larvae (9 d post-treatment)	27.2 (1.02–5.15)	
Adult (1 × 10^7^ spore/mL)		8.8 (7.8–10.2)
Adult (1 × 10^8^ spore/mL)		4.9 (4.1–5.8)
2nd instar larvae (1 × 10^8^ spore/mL)		9.3 (8.1–11.7)
2nd instar larvae (2 × 10^8^ spore/mL)		4.6 (3.8–5.5)

The insect soaking method was used to conduct the adult bioassay; the anesthetized CFBs with CO_2_ were moved into the tubes filled with conidial suspension with a designated conidial concentration and immersed for 20 s, fed with fresh CFC leaves, and placed in an artificial climate chamber at 26 ± 1 °C with a photoperiod of 14:10 h (light:dark) and 100% humidity. The experiment was replicated 3 times, and 10 insects in each treatment were used. For the larvae bioassay, first, the radish (*R. sativus*) pieces were dipped in the conidial solution for 10 s and dried. Afterward, larvae (n = 10) were moved onto the radish pieces and placed in an artificial climate chamber at 26 ± 1 °C in the dark, and the old radish pieces were substituted with fresh ones every day. There were 10 insects in each treatment and the experiments were repeated 4 times. After the treatments, the diseased insects were determined based on the presence of the fungal colony on the cadaver. The LC_50_s are from the data at 9 d post-treatment, while LT_50_s represent the results under certain conidial concentrations.

**Table 2:** Effects of MaGX19S02 pelletized seeds on CFB and CFC in the pot test.

Conidia Dose (mg/g Seed)	Corrected Mortality of CFB Larvae (%)	CFC Seedling Rate (%)	CFC Plant Height (cm)
12.5	45.45 ± 0.08 b	67.50 ± 5.42 b	7.70 ± 0.50 a
25	72.73 ± 0.08 a	78.33 ± 6.11 ab	8.40 ± 0.60 a
50	81.82 ± 0.05 a	86.67 ± 6.38 a	8.80 ± 0.70 a
0 (CK)		43.33 ± 3.15 c	5.40 ± 0.50 b

Data obtained on day 20 post-sowing; different letters in columns represent a significant difference (*p* < 0.05). The experiment was repeated t3 times with 30 seeds and 10 1st instar larvae in each pot. is the results indicate that Ma pelletized seeds can significantly control CFB larvae and raise CBC seedling rate and plant height.

**Table 3:** Damage index (DI) and control efficacy (CE) of pelletized CFC seeds on CFB in the field.

Seed	Conidia (mg/g Seed)	7 d Post-Sowing		10 d Post-Sowing		14 d Post-Sowing	
		DI	CE (%)	DI	CE (%)	DI	CE (%)
Ma pelletized	12.5	5.2 ± 0.1 c	60.5 ± 2.3 b	10.0 ± 0.8 b	41.3 ± 2.7 b	15.9 ± 1.1 b	57.0 ± 3.3 b
	25.0	2. 2 ± 0.0 c	83.1 ± 5.5 a	5.9 ± 0.4 b	65.2 ± 4.2 ab	12.0 ± 0.9 b	67.5 ± 3.7 ab
	50.0	1.9 ± 0.0 c	92.9 ± 6.2 a	4.8 ± 0.4 b	85.8 ± 5.4 a	14.3 ± 1.2 b	80.6 ± 4.3 a
Filler pelletized	0.0	8.7 ± 0.1 b	33.8 ± 2.1 c	18.2 ± 1.5 a		40.7 ± 2.8 a	
Normal seeds	0.0	13.2 ± 0.1 a		17.0 ± 1.3 a		37.0 ± 2.6 a	

The data were from two field trials conducted at the South China Agricultural University (Guangzhou, China) farm in September–November 2020 and February–April 2021, following a random block design with three replicates. Each plot (20 m^2^) was sown with 40 g of pelletized or 20 g of normal seeds. The efficacy damage index of CFC was estimated referring to the China National Standards (GB/T 17980.18). Different letters in the columns indicate significant differences (*p* < 0.05) in the DMRT.

**Table 4:** Effects of chemical insecticides on MaGX19S02.

Treatment	Conidia Germination (%)		Mycelia Growth (Colony Diameter, mm)			
			Post-Treatment 7 d		Post-Treatment 14 d	
	10 mg/L	100 mg/L	10 mg/L	100 mg/L	10 mg/L	100 mg/L
Acetamiprid	80.22 ± 0.01 c	80.37 ± 0.05 c	33.3 ± 0.3 a	32.7 ± 0.3 ab	61.3 ± 0.3 ab	62.3 ± 0.3 b
Chlorfenapyr	82.53 ± 0.06 c	86.68 ± 0.04 a	33.0 ± 0.6 a	35.3 ± 0.3 a	62.3 ± 1.5 a	66.7 ± 0.3 a
Diafenthiuron	87.12 ± 0.04 b	87.06 ± 0.05 a	33.0 ± 0.6 a	32.0 ± 0.6 b	63.0 ± 1.0 a	55.7 ± 1.2 d
Dinotefuran	81.79 ± 0.06 c	80.23 ± 0.02 c	31.0 ± 1.5 b	27.3 ± 0.3 c	61.3 ± 1.8 ab	63.6 ± 1.5 b
Emamectin benzoate	76.07 ± 0.06 d	84.68 ± 0.01 b	34.0 ± 0.0 a	32.7 ± 0.3 ab	60.3 ± 1.2 bc	61.7 ± 0.9 bc
Pyridaben	83.44 ± 0.06 c	82.09 ± 0.05 c	32.0 ± 0.0 ab	30.7 ± 1.2 b	60.3 ± 1.5 bc	59.3 ± 1.2 c
Rotenone	69.73 ± 0.01 e	82.35 ± 0.04 c	32.0 ± 1.0 ab	30.7 ± 0.7 b	56.7 ± 0.9 d	61.7 ± 0.3 bc
Thiamethoxam	74.35 ± 0.02 d	88.48 ± 0.04 a	33.7 ± 0.3 a	34.7 ± 0.7 a	62.7 ± 1.5 a	65.7 ± 1.5 a
Tolfenpyrad	91.75 ± 0.02 a	86.96 ± 0.01 a	30.3 ± 0.3 b	27.0 ± 0.6 c	59.7 ± 0.3 c	49.0 ± 0.6 e
CK	88.70 ± 0.02 b	88.12 ± 0.02 a	33.0 ± 1.2 a	33.0 ± 1.2 ab	59.7 ± 0.9 c	59.7 ± 0.9 c

Data show the mean ± SD of three replicates. Different letters indicate significant differences (*p* < 0.05) in the DMRT; CK, the acetone solution (100 mg/L) with Tween-80 (0.05%). The effects of these insecticides on Ma conidia germination and mycelial growth in shaking culture and PDA plate were investigated (referring GB/T 25864). The insecticides were dissolved into 10,000 mg/L (stock solution) with acetone and diluted with 0.05% Tween-80 (working solutions). The experiment was repeated three times.

**Table 5:** Joint virulence of MaGX19S02 and chlorfenapyr against *P. striolata* adults.

A/B (*v*/*v*)	a.i. (mg/L)		LT_50_ (95% Confidence Interval) (d)	CTC
	MaGX19S02	Chlorfenapyr		
1/9	42.4	45	6.66 (5.68–7.69)	85.60
3/7	127	35	6.98 (5.90–8.19)	94.02
5/5	212	25	5.19 (4.31–5.99)	151.59
7/3	296	15	4.82 (3.86–5.68)	204.18
9/1	381	5	6.26 (5.53–6.98)	209.86
0/10	0	50	8.93 (7.93–10.33)	
10/0	424	0	9.73 (8.68–11.33)	

A, MaGX19S02 10^10^ spores/L (424 mg/L); B, 50 mg/L chlorfenapyr; a.i., content of active ingredients. When CTC > 120, the mixture has synergism. is the results indicate that A/B rates (*v*/*v*) of 5/5, 7/3, and 9/1 are synergistic. The insect soaking method was used. The experiments were repeated 3 times with 10 insects in each treatment.

**Table 6:** CFB mortality after treatment in pot test.

Treatment				Mortality (%)		
Insecticide		a.i. (mg/L)		Post-Treatment 3 d	Post-Treatment 5 d	Post-Treatment 7 d
		Ma	Chlorfenapyr			
20% Ma-Chl WP	500×	380	20	23.33 ± 3.33 a	56.67 ± 3.33 a	93.33 ± 6.67 a
	1000×	190	10	20.00 ± 3.33 a	40.00 ± 5.77 ab	50.00 ± 3.33 b
	1500×	126.3	6.7	6.67 ± 3.33 a	20.00 ± 5.77 bc	40.00 ± 5.77 b
	2000×	95	5	0 b	16.67 ± 6.67 bc	30.00 ± 6.67 bc
	3000×	63.7	3.3	0 b	10.00 ± 5.77 bc	23.33 ± 3.33 bc
Chlorfenapyr			50	10.00 ± 5.77 a	23.33 ± 3.33 bc	26.67 ± 6.67 bc
MaGX19S02		424 *		0 b	10.00 ± 5.77 bc	33.33 ± 3.33 bc
CK		0	0	0 b	0 c	3.33 ± 3.33 c

Notes: Data show the mean ± SD; different letters in different columns indicate significant differences (*p* < 0.05) in the DMRT, * 424 mg is 1 × 10^10^ spores/L; a.i., content of active ingredients. A total of 50 seeds were sowed in each pot. After 20 days of culture, each pot was sprayed with different concentrations of Ma-Chl WP (10 mL). Then, CFB adults (n = 10) were released into each pot, and the pots were covered with a nylon mesh to prevent insect escape. Tap water was used as a control. The mortality rate of adults and the damage index (DI) of the CFC were assessed according to the China National Standards (GB/T 17980.18). The experiment was repeated three times.

**Table 7:** Damage index (DI) and control efficacy (CE) of Ma-Chl WP in field trial.

Treatments		Post-Treatment 7 d		Post-Treatment 14 d	
Insecticide	a.i. (mg/L)	DI	CE (%)	DI	CE (%)
20% Ma-Chl WP 500×	400	19.4 ± 1.4 c	61.3 ± 4.5 *	24.4 ± 1.3 b	56.2 ± 5.1 *
Chl	50	56.7 ± 4.0 b	24. 8 ± 2.1	84.4 ± 2.6 a	−0.9 ± 0.5
CK	0	80.0 ± 5.7 a		88.9 ± 3.4 a	

Notes: a.i., content of active ingredients. * indicates significant differences in the T-test. Different letters in the DI columns indicate significant differences (*p* < 0.05) in the DMRT. The field trials were conducted at the South China Agricultural University (Guangzhou, China) farm during September–November 2020 and February–April 2021, following a random block design with three replicates. Each plot (20 m^2^) was sown with 20 g of normal seeds and sprayed with 1 L of diluent when the CFC adult density was ~1/plant. The plot was covered with nylon mesh after spraying. The efficacy was evaluated based on the damage index of the CFC referring to the China National Standards (GB/T 17980.18).

## Data Availability

The data used in this analysis were obtained upon request from the cited publisher. The data are available upon request from the corresponding author.

## Supplementary Materials

The following supporting information can be downloaded at: https://www.mdpi.com/article/10.3390/insects14060567/s1, Table S1: Bioactivity of different fungal strains against *Phyllotreta striolata*.

## Abbreviations

BCA	Biological control agent
CE	Control efficacy
CFB	Cabbage flea beetle (*Phyllotreta striolata*)
CFC	Chinese flowering cabbage (*Brassica campestris* L. ssp. *chinensis* var. *utilis* Tsenet Lee)
Chl	Chlorfenapyr
DI	Damage index
EF	Entomopathogenic fungi
Ma	Metarhizium anisopliae strain MaGX19S02
Ma-Chl WP	MaGX19S02 and chlorfenapyr mixed wettable powder
WP	Wettable powder
