# Supplementary material for: Screening of Fungal Strains and Formulations of Metarhizium anisopliae to Control Phyllotreta striolata in Chinese Flowering Cabbage
Source: Insects. 2023 Jun 19;14(6):567. doi: 10.3390/insects14060567 (PMC10299718; doi:10.3390/insects14060567)
Supplement: Supplementary file 1 [file insects-14-00567-s001.zip › insects-2373860-supplementary materials.pdf]

insects

Supplementary Materials

Table S1. Bioactivity of different fungal strains against *Phyllotreta striolata.*

Fungi species	Number	Corrected Mortality (%)		
		7 d	9 d	11 d
Beauveria bassiana	BbGX22A02	6.67 ± 0.09	6.67 ± 0.09	6.67 ± 0.09
	BbGX6502	0.00 ± 0.00	0.00 ± 0.00	0.00 ± 0.00
	BbGX7303	6.67 ± 0.09	6.67 ± 0.09	40.00 ± 0.28
	BbGX10A01	10.00 ± 0.08	13.33 ± 0.05	26.67 ± 0.09
	BbGX7703	6.67 ± 0.09	20.00 ± 0.28	20.00 ± 0.28
	BbGX77A01	13.33 ± 0.09	13.33 ± 0.09	40.00 ± 0.16
B. brongniartii	BbGX70A02	0.00 ± 0.00	0.00 ± 0.00	0.00 ± 0.00
Cordyceps fumosorosea	IfGX20A02	0.00 ± 0.00	6.67 ± 0.09	6.67 ± 0.09
	IfGX21G01	0.00 ± 0.00	0.00 ± 0.00	0.00 ± 0.00
	IfGX2802	0.00 ± 0.00	6.67 ± 0.09	6.67 ± 0.09
	IfGX21L01	13.33 ± 0.09	13.33 ± 0.09	13.33 ± 0.09
	IfGX33H05	0.00 ± 0.00	26.67 ± 0.25	26.67 ± 0.25
	IfGX30S01	6.67 ± 0.09	6.67 ± 0.09	20.00 ± 0.16
	IfGX30A01	0.00 ± 0.00	13.33 ± 0.19	20.00 ± 0.16
	IfGX70A01	13.33 ± 0.09	13.33 ± 0.09	13.33 ± 0.09
	IfGX7704	6.67 ± 0.05	13.33 ± 0.05	16.67 ± 0.05
	IfGZ4304	6.67 ± 0.09	6.67 ± 0.09	13.33 ± 0.09
	IfGX2303	13.33 ± 0.19	13.33 ± 0.19	13.33 ± 0.19
C. *farinosa*	IfGX6701	0.00 ± 0.00	0.00 ± 0.00	0.00 ± 0.00
C. *cateniannulata*	IcGX32S01	0.00 ± 0.00	26.67 ± 0.09	26.67 ± 0.09
Metarhizium anisopliae	MaYN8304	20.00 ± 0.04	30.00 ± 0.08	38.33 ± 0.13
	MaGX33H02	6.67 ± 0.09	6.67 ± 0.09	6.67 ± 0.09
	MaGX7002	0.00 ± 0.00	6.67 ± 0.09	6.67 ± 0.09
	MaGX23A01	0.00 ± 0.00	0.00 ± 0.00	6.67 ± 0.09

	MaGX19S02	46.67 ± 0.09	46.67 ± 0.09	53.33 ± 0.09
	MaGX13S01	0.00 ± 0.00	0.00 ± 0.00	6.67 ± 0.09
	MaGX19J01	0.00 ± 0.00	6.67 ± 0.09	6.67 ± 0.09
	MaGX0601	0.00 ± 0.00	20.00 ± 0.28	20.00 ± 0.28
	MaGX7201	13.33 ± 0.09	20.00 ± 0.16	20.00 ± 0.16
	MaGX02A02	0.00 ± 0.00	0.00 ± 0.00	3.33 ± 0.05
	MaGX09A02	3.33 ± 0.05	10.00 ± 0.08	20.00 ± 0.16
	MaYN8304	20.00 ± 0.09	30.00 ± 0.09	38.33 ± 0.09
	MaGX7702	6.67 ± 0.09	20.00 ± 0.00	20.00 ± 0.00
M. guizhouense	MgGX1103	0.00 ± 0.00	13.33 ± 0.09	23.33 ± 0.17
M. flavoviride	MfGX6501	6.67 ± 0.09	6.67 ± 0.09	13.33 ± 0.09
	MfGX33Y01	0.00 ± 0.00	0.00 ± 0.00	6.67 ± 0.09
Pochonia	P.GX0801	6.67 ± 0.09	16.67 ± 0.13	26.67 ± 0.21
P.chlamydosporia	PcGX13Z02	0.00 ± 0.00	6.67 ± 0.09	6.67 ± 0.09
	PcGX2301	13.33 ± 0.09	13.33 ± 0.09	13.33 ± 0.09
	PcGX33Y03	0.00 ± 0.00	0.00 ± 0.00	0.00 ± 0.00
	PcGX08A01	10.00 ± 0.00	13.33 ± 0.05	20.00 ± 0.08
P. bulbillosa	PbGX13S04	0.00 ± 0.00	13.33 ± 0.12	13.33 ± 0.12
	PbGX32S02	0.00 ± 0.00	6.67 ± 0.09	6.67 ± 0.09
	PbGX16S04	0.00 ± 0.00	13.33 ± 0.19	13.33 ± 0.19
Purpureocillium lilacinum	PlYN6601	0.00 ± 0.00	6.67 ± 0.09	6.67 ± 0.09
	PlGX7402	0.00 ± 0.00	0.00 ± 0.00	0.00 ± 0.00
	PlGX7706	6.67 ± 0.09	13.33 ± 0.09	13.33 ± 0.09
	PlGX1001	6.67 ± 0.09	6.67 ± 0.09	6.67 ± 0.09
	PlGX77A04	6.67 ± 0.09	6.67 ± 0.09	6.67 ± 0.09
	PlGX0802	3.33 ± 0.05	6.67 ± 0.05	13.33 ± 0.09
	PlGX06A01	6.67 ± 0.05	10.00 ± 0.05	13.33 ± 0.05
	PlGX7202	0.00 ± 0.00	0.00 ± 0.00	0.00 ± 0.00

	PlGX6802	0.00 ± 0.00	0.00 ± 0.00	6.67 ± 0.09
	PlGX70A03	0.00 ± 0.00	0.00 ± 0.00	0.00 ± 0.00
	PlGX17A01	0.00 ± 0.00	0.00 ± 0.00	0.00 ± 0.00
	PlYN0401	6.67 ± 0.09	13.33 ± 0.09	23.33 ± 0.17
	PlYN0203	15.00 ± 0.15	20.00 ± 0.19	28.33 ± 0.27
	PlGX16S03	6.67 ± 0.09	20.00 ± 0.16	3.33 ± 0.25
	PlGX1702	20.00 ± 0.16	20.00 ± 0.16	20.00 ± 0.16
	PlGX6901	0.00 ± 0.00	0.00 ± 0.00	0.00 ± 0.00
	PlGX7101	13.33 ± 0.09	13.33 ± 0.09	13.33 ± 0.09
	PlGX7003	26.67 ± 0.25	26.67 ± 0.25	26.67 ± 0.25
	PlGX04A01	6.67 ± 0.05	10.00 ± 0.08	16.67 ± 0.09
	PlGX0501	0.00 ± 0.00	3.33 ± 0.05	6.67 ± 0.09
	PlGX0401	6.67 ± 0.05	13.33 ± 0.05	23.33 ± 0.05
	PlGX0201	20.00 ± 0.28	20.00 ± 0.28	26.67 ± 0.25
	PlGX19S01	0.00 ± 0.00	0.00 ± 0.00	0.00 ± 0.00
	PlYN6701	6.67 ± 0.09	33.33 ± 0.09	40.00 ± 0.28
	PlGX7302	0.00 ± 0.00	0.00 ± 0.00	0.00 ± 0.00
	PlYN5901	6.67 ± 0.09	6.67 ± 0.09	13.33 ± 0.09
	PlGZ5801	0.00 ± 0.00	13.33 ± 0.19	26.67 ± 0.25
	PlYN8102	20.00 ± 0.28	33.33 ± 0.34	33.33 ± 0.34
	PlYN8002	6.67 ± 0.09	20.00 ± 0.16	20.00 ± 0.16
	PlYN8202	26.67 ± 0.09	26.67 ± 0.09	33.33 ± 0.09
P. lavendulum	PlGZ4402	0.00 ± 0.00	0.00 ± 0.00	0.00 ± 0.00
	PlGX6801	5.00 ± 0.00	18.33 ± 0.05	30.00 ± 0.07
	PlGX6503	0.00 ± 0.00	0.00 ± 0.00	6.67 ± 0.09
Paecilomyces marquandii	PmGX7701	0.00 ± 0.00	0.00 ± 0.00	0.00 ± 0.00
	PmGX12Y01	0.00 ± 0.00	0.00 ± 0.00	6.67 ± 0.09
	PmGX7001	0.00 ± 0.00	0.00 ± 0.00	6.67 ± 0.09

	PmGX24G02	0.00 ± 0.00	0.00 ± 0.00	0.00 ± 0.00
	PmGX33H03	6.67 ± 0.09	20.00 ± 0.16	20.00 ± 0.16
	PmYN8303	6.67 ± 0.09	6.67 ± 0.09	20.00 ± 0.28
	PmGX7301	0.00 ± 0.00	0.00 ± 0.00	6.67 ± 0.09
	PmGX6506	0.00 ± 0.00	0.00 ± 0.00	0.00 ± 0.00
Paecilomyces	P.YN0202	5.00 ± 0.00	6.67 ± 0.02	6.67 ± 0.02
	P.GX77A03	6.67 ± 0.09	13.33 ± 0.19	13.33 ± 0.19
	P.GX78A01	0.00 ± 0.00	6.67 ± 0.09	13.33 ± 0.09
P. carneus	PcYN8101	13.33 ± 0.09	13.33 ± 0.09	13.33 ± 0.09
	PcYN7801	6.67 ± 0.09	13.33 ± 0.09	20.00 ± 0.00
	PcYN8001	6.67 ± 0.09	13.33 ± 0.09	13.33 ± 0.09
Paecilomyces	P.GX6505	0.00 ± 0.00	6.67 ± 0.09	6.67 ± 0.09
P. citrinum	PcGX1605	6.67 ± 0.09	20.00 ± 0.16	20.00 ± 0.16
	PcGX21L02	6.67 ± 0.09	13.33 ± 0.09	13.33 ± 0.09
	PcGX78A02	0.00 ± 0.00	6.67 ± 0.09	6.67 ± 0.09
Acremonium	A.YN0403	11.67 ± 0.08	33.33 ± 0.05	46.67 ± 0.06
Doratomyces	D.GX11G02	3.33 ± 0.05	3.33 ± 0.05	3.33 ± 0.05
Lecanicillium	L.GZ5601	0.00 ± 0.00	0.00 ± 0.00	0.00 ± 0.00
Mortierella	M.GX13S05	0.00 ± 0.00	13.33 ± 0.09	20.00 ± 0.00
Nectria	N.GX2905	8.33 ± 0.08	16.67 ± 0.10	23.33 ± 0.14
Scopulariopsis brumptii	SbGX7705	10.00 ± 0.00	20.00 ± 0.08	30.00 ± 0.14
Talaromyces	T.GX3101	0.00 ± 0.00	13.33 ± 0.12	13.33 ± 0.12
	T.GX05A01	0.00 ± 0.00	0.00 ± 0.00	6.67 ± 0.05

The fungal strain concentration: 1×10^7 spores/mL. The mortality data were mean ± SE. The numbers of insects for test were 10 in each treatment. The experiment was repeated three times.
